# A List of the Most Important Works, Essays, Cases, and Facts, Connected with Medical Science

**Published:** 1826-01

**Authors:** 


### A LIST

OF THE MOST IMPORTANT

### WORKS, ESSAYS, CASES, AND FACTS,

### CONNECTED WITH MEDICAL SCIENCE,

*Which have either been originally published in this Country or noticed in the Medical Journals, during the year* 1825;

ARRANGED ACCORDING TO SUBJECTS.*

---

### ANATOMY, *in general.*

Elements of the Anatomy of the Human Body, in its sound State, &c. &c.; by Dr. A. Munro.—Reviewed in the Edinburgh Medical and Surgical Journal, No. lxxxv.

Manuel d'Anatomie Generale, Descriptive et Pathologique, par Prof. Meckel; traduit et augmentée par A. J. L. Jourdan et Prof. Breschet.—Reviewed in this Journal, March, April, May, and June.

Introductory Lectures on Anatomy, by Dr. Baillie. (Printed, but not published.) London, 1825.

A System of Anatomical Plates, by Mr. Lizars, up to Part VIII. Edinburgh.

A Course of Dissections for the use of Students, by Mr. H. Mayo. London, 1825.

A Manual of Anatomy, by Dr. W. Bennett. Edinburgh, 1825.

*Anatomy of particular Parts.*

Illustrations of the Arteries connected with Aneurism and Surgical Operations, by Mr. Dermott. London, 1825.

Surgical Anatomy of the Arteries of the Human Body, by Mr. Harrison. Dublin, 1825.

Anatomie du Cerveau, contenant l'Histoire de son Developpement dans le Fœtus, &c. par F. Tiedemann; traduit de l'Allemand, par A. J. L. Jourdan.—Reviewed in Edinburgh Medical and Surg. Journal, January; and translated into English by Dr. Bennett, Edinburgh.

On the Ligaments of the Human Ossicula Auditus, by Mr. Chevalier.—Medico-Chirurgical Transactions, vol. xiii. part i.

On Ligaments, by Mr. B. Cooper. London.

A Short Description of the Bones, &c. by Mr. South. London, 1825.

Discovery of a Lymphatic Vessel opening into the Vena Cava inferior.—Bulletin des Sciences Medicales, Dec. 1824. This Journal, March 1825.

Note on the Structure of the Nerves, by M. Bogros.—Revue Medicale, Mai; Archives Generales, Juin; this Journal, September; Med. Repos. July; Lancet, July 30; Anderson's Journal, October.

A Practical Treatise on the Arterial System, by Mr. Turner. London, 1825.

---

* We have adopted the above-mentioned limitations, as extending the list to the productions of foreign countries would have rendered it greatly more extensive. We do not profess to have enumerated *all* the Works or Cases which have been published, but trust that the *most important* will be found in our selection. Where a work has been reviewed in various Journals, or a fact mentioned, we have endeavoured to give reference to these according to the dates of their appearance; and, where any omission may be found, we request the respective Authors and Editors to believe that it has resulted from oversight, not design.—Editors.

Lachrymal Nerve, said to be a Branch of the Fourth, by M. Amusat.—Medical Repository, December.

On the different Appearances presented by the Mucous Membrane of the Stomach and Intestinal Canal, in a healthy State, by M. Rousseau.—Archives Generales, November; Lancet, January 15 and 22.

*Anatomy, Comparative.*

Memoir on the Bag or Bladder in the Mouth of the Dromedary, by Dr Paoli Savi.—Edinburgh Philosophical Journal, Jan. and April.

On the Cochlea of the Internal Ear of Birds, by Dr. G. R. Treviranus.—Edinburgh Philos. Journal, January; this Journal, March.

Note on the Spider whose Web is employed in Medicine.—Journal of the Academy of Natural Sciences, Philadelphia, vol. ii.; Edinburgh Philos. Journal, January.

On the Existence of the Pancreas in some of the Cuttle Fish Tribe, and the Doris Argo, by Dr. Grant.—Edinburgh Philos. Journal, July.

Part Second of a Series of Elementary Lectures on the Anatomy, &c. of the Horse, by Mr. Percival. London, 1825.

---

### PHYSIOLOGY.

*Physiology in general.*

Elements of Physiology, by Dr. Rudolphi, of Berlin; translated from the German, by Dr. How. Vol. i. London, 1825.

Conversations on Physiological Medicine; translated from the French. London, 1825.

Observations Pathologiques propres a eclairer plusieurs points de Physiologie, par M. Lallemand.—Analysed in this Journal, March.

*Physiology of the Nervous and Sensitive Systems.*

Observations on the Effects of dividing the Eighth Pair of Nerves, by Dr. W. Phillip.—Medical and Chirurgical Review, October.

M. Defermon on ditto.—Anderson's Quarterly Journal, October.

Case illustrating the Semidecussation of the Optic Nerves, by Dr. Crawford.—This Journal, January.

New Theory of Vision, by R. Lehot.—Revue Medicale, Decembre 1824; Lancet, February 26.

On the Motions of the Eye, by Mr. Charles Bell; read before the Philosophical Society of Edinburgh, March 1825.—Edinb. Phil. Journal, April.

On the Existence of Nerves in the Placenta, by Sir E. Home, Bart.—Philosophical Transactions, 1825, part ii.

Experiments made upon six decapitated Robbers, by Prof. Bartels.—Schreften der Gesell der Gerammt Naturwiss zu Marburg, vol. i.; and this Journal, February.

On Sympathy, by Mr. Jones.—This Journal, March, &c.

On the Degree of Sensibility possessed by the Nerves of the Senses, by M. Magendie.—Bulletin des Sciences Medicales, Mars; this Journal, May and June.

On the Insensibility of the Retina, by M. Magendie.—Journal de Physiologie, Juin; Lancet, August 6.

On the Functions of certain Parts of the Nervous System, by M. Magendie.—Lancet, April 9.

Experimenta in Nervorum Antagonismum Natura, a Carolo F. Bellingeri.—Lancet, May 7.

*Physiology of the Circulating System.*

Remarks on the asserted Muscularity of Arteries, by Mr. Mackenzie, of Glasgow.—This Journal, January.

On the Motion of the Heart, by Mr. Alderson.—Anderson's Quarterly Journal, January.

Experiments to determine whether the Blood in the Veins be moved by a Suction Power of the Heart, by Mr. Paterson.—Edinburgh Medical and Surgical Journal, No. lxxxiv.

Syphonic Theory; or, Brief Observations on the Circulation of the Blood, and on Respiration as connected therewith, by Mr. Hopley. London, 1825.

Memoir on the Motion of the Blood in the Veins, by Dr. Barry.—Medical Repository, October.

On the Quantity of Blood in Animals, by Dr. Kidd.—Edinburgh Philos. Journal, April.

A Short Inquiry into the Capillary Circulation of the Blood, by Dr. Black. London, 1825.

Experiments on the Injection of Air into the Veins, by MM. Ficinus and Prinz, of Dresden.—Anderson's Quarterly Journal, October.

*Physiology of the Absorbent System.*

Case in which Chyle was found in the Veins of the Jejunum, by Dr. Meyer, of Bonn.—Journal Complementaire, Aout; this Journal, Nov.

Of the present State of our Knowledge respecting the Function of Absorption.—Medical Repository, January and March.

Experiments on the Direct Passage of Substances into the Blood, by M. Westrumb.—Anderson's Quarterly Journal, July.

*Physiology of the Respiratory System and Calorification.*

On Asphyxia by Strangulation, by M. Segalas.—Edinburgh Philosophical Journal, January.

On the Causes of Animal Heat, by M. Despretz.—Annales de Chimie, No. xxvi.; Edinburgh Philos. Journal, April.

Observations on the Temperature of Man and other Animals, by Dr. Davy.—Edinburgh Philos. Journal, October.

*Physiology of the Digestive System.*

On the Digestive Process, by MM. Prevost and Royer.—Archives Generales; Lancet, September 3.

*Physiology of the Urinary System.*

Chemical Examination of the Changes which Substances undergo in their Passage through the Circulation, and their Excretion by the Kidneys. —Journal der Physiologie, von Tiedemann; Lancet, June 18 and Nov. 26.

*Physiology of Generation.*

New Theory of Generation, by Professor Rolando.—Revue Med. Mars Lancet, July 30; Medical Repository, August; and Anderson's Quarterly Journal, October.

*Miscellaneous Points of Physiology.*

On Instinct, by Dr. Mason Good.—This Journal, November: reviewed in the Lancet, November 26.

---

### MORBID ANATOMY.

*Morbid Anatomy in general.*

Baillie's Morbid Anatomy; to which are prefixed, Preliminary Observations on diseased Structures, by Mr. Wardrop: being Vol. ii. of the Works of Matthew Baillie, M.D. London, 1825.—Reviewed in the Lancet, Oct. 29.

*Morbid Anatomy of the Brain and Nerves.*

Observations on the Malformation of the Brain and Nerves, by M. Tiedemann.—Zeitung für Physiologie, &c.; Lancet, August 20.

Case of diseased Spinal Marrow.—Archives Generales, January; this Journal, March.

Case of Fungus Hæmatodes of the Brain, by Mr. Hunter.—Medico-Chirurgical Transactions, vol. xiii. part i.

Case of Hæmatoid Tumor of the Brain, by Dr. G. Gregory.—This Journal, December.

Tuberculous Affection of the Brain, with Destruction of the Ethmoidal Nerves, by M. Desmoulins.—Journal de Physiologie, Avril; this Journal, September; Medical Repository, September.

Case of Tubercular Disease of the Brain, with Destruction of the Olfactory Nerves, by M. Berard.—Journal de Physiologie, January; Anderson's Quarterly Journal, October.

Case of Bifid Brain, with Hydrocephalus, by Dr. Spurzheim.—Phrenological Journal, vol. ii. No. 7.

*Morbid Anatomy of the Heart and Arteries.*

Case of Enlargement of the Heart, by Dr. Howison.—Edinburgh Med. and Surgical Journal, July.

Case of Rupture of the Right Auricle of the Heart, by Mr. W. Thomas.—This Journal, March.

Cases of Aneurism of the Heart, by M. Bricheteau.—Journal Complementaire; Med. and Chirurgical Review, July.

Case of Cancer of the Heart, by M. Legalas.—Archives Generales, September; this Journal, December.

Case of Rupture of the Heart, by M. Ferrus.—Revue Medicale, Aout; this Journal, November.

Singular Ossification of the Heart.—Rev. Med. Aout; this Journal, Nov.

Case of Rupture of the anterior Portion of the left Ventricle of the Heart, by M. Carrier.—Journal Universel, Sept. 1824; Med. Repos. Jan. 1825.

Case of Tympanitis of the Pericardium.—Med. and Chir. Review, April.

Case of Inflammation of the Pericardium, by M. Bouillaud.—Archives General, February; Medical and Chir. Review, July.

Case of Aneurism of the Aorta, opening into the Spinal Canal.—Archives Generales, May; Lancet, July 9.

Case of Aneurism of the anterior Cerebral Artery, by Mr. Spurgin.—Medical Repository, June.

*Morbid Anatomy of the Lymphatic System.*

Cases of Inflammation, Obliteration, Cancer, &c. of the Thoracic Duct, by M. Andral.—Revue Medicale; Medical Repository, February and March; Lancet, February 5; Anderson's Quarterly Journal, April.

*Morbid Anatomy of the Alimentary Canal and adjacent Viscera.*

Case of Perforation of the Stomach, by M. Martinet.—Revue Medicale, January; Medical and Chir. Review, July.

Morbid Appearances observed in the Stomach in Phthisis Pulmonalis, by M. Andral.—Revue Medicale, April; Medical Repository, July.

Softening of the Stomach in sixteen Cases of Hydrocephalus, by Dr. Blackhausen, of Bremen.—Bib. der Prak. Heilk.; Med. Repos. August.

Two Cases of Softening of the Stomach, by M. Ferrus.—Revue Medicale, Août; this Journal, November.

Case of Intestinal Calculus, by Mr. Torbet.—Edinburgh Medical and Surgical Journal, July.

Case in which two Fish-bones were found imbedded in the Duodenum, by M. Hervey.—Archives Generales, May; Lancet, July 9.

Account of an immense Gall-stone.—Lancet, December 3.

Case of Ossification of the Spleen.—Rust's Magazin für die Gesammt Heilkunde; this Journal, September.

Case of Hydatid Ascites, by Dr. Long.—This Journal, August.

Case in which the Epiploon was found Dropsical, in a Fœtus of eight Months.—Archives Generales, July; This Journal, October.

*Morbid Anatomy of the Urinary System.*

Case in which 678 Calculi were found in the Bladder of a Man, by M. Murat.—Revue Medicale, Juin; this Journal, October.

*Morbid Anatomy of the Generative System.*

Case of Ossification of the Uterus, by Mr. Fowkes.—This Journal, Dec.

Case of Fungoid Tumor of the Uterus, by Dr. Crowfoot.—Edinburgh Medical and Surgical Journal, January.

Case of Double Uterus, with Rupture of that Portion containing the Fœtus, by M. Olivier.—Archives, Generales; Lancet, September 3.

Case of Malformation of the Organs of Generation.—Lancet, July 23.

*Morbid Anatomy of the Bones.*

Unnatural Growth of the Bones of the Cranium, by M. Devergie.—Revue Medicale, Avril; this Journal, June.

Case of complete Anchylosis of the Joints.—Annali Universali; Lancet, April 9.

*Miscellaneous Points in Morbid Anatomy.*

Case of a Child who was born wanting the left Foot, which had been removed in Utero, as if amputated.—This Journal, July.

Case of Tumor, containing Bone and Teeth, found in the anterior Mediastinum, by Dr. Gordon.—Medico-Chir. Transactions, vol. xiii. part i.

---

### PATHOLOGY.

*Pathology in general.*

Collections from the unpublished Medical Writings of the late Dr. Parry; Introductory Essays, by Dr. C. H. Parry. London, 1825.—Reviewed in Medical Repository, May; Anderson's Journal, October.

Elements of Pathology and Therapeutics, by Dr. C. H. Parry. London, 1825.—Analysed in Lancet, May 7; Medical and Chir. Review; and Anderson's Journal, July.

An Inquiry into the Seat and Nature of Fever, by Dr. Clutterbuck. Second Edition. London, 1825.

Pathology of various Diseases, by Mr. Jones.—This Journal, Feb. &c.

Remarks on Irritative Fever, by Dr. Butter.—Analysed in the Medical Repository, October and December; and in this Journal, Sept.

Clinique Medicale, &c. par M. Andral. Premiere partie, Fievres. Paris, 1823.—Edinburgh Med. and Surgical Journal, January 1825.

Observations on the Humoral Pathology, by Dr. Stoker.—This Journal, May.

Case of Irritative Fever, arising from a Scratch received in a morbid Dissection, by Dr. A. T. Thomson.—Medical Repository, April; and this Journal, May.

Cases of the Fatal Disease which occurred, during last Autumn, at the Dock-yard at Devonport, by Mr. Tripe.—This Journal, September.

*Pathology of the Brain and Sentient Systems.*

Outlines of Lectures on Mental Diseases, by Dr. A. Morison. Edinburgh, 1825.

On Mental Alienation, with fifty Cases in which the Brain was examined after Death, by M. Neumann, Berlin.—Medical Repository, Feb. &c.

On the Pathology and Treatment of Headache, by Dr. I. L. Morgan.—Edinburgh Medical and Surgical Journal, Nos. lxxxiv. and v.

Essay on the Nature, Causes, and Treatment, of Hydrocephalus, by Dr. Shearman.—Analysed in this Journal, and in the Med. Repository, Dec.

On Enlargement or Hypertrophy of the Brain, by M. Hufeland.—Bull. des Sciences Medicales, Dec. 1824; this Journal, March 1825; Lancet, March 26.

——————————————, by M. Scutetten.—Archives Generales, Jan.; this Journal, March; Lancet, March 26.

Clinical Observations, tending to prove that Loss of Speech, in many Cases, depends upon a Lesion of Part of the anterior Lobes of the Brain, by M. Bouillaud.—Archives Generales; Lancet, July 23.

On the Hallucinations of the Senses, by M. Bayle.—Revue Medicale, January; Lancet, March 5 and 12.

New Doctrine of Mental Diseases, by M. Bayle.—Analysed in Lancet, April 23.

On the Seat and Cause of Epilepsy, by M. Alphonse Menard.—Revue Medicale, March; Lancet, June 4; Med. and Chir. Review, July.

Observations on a peculiar Species of Convulsions in Children, by Mr. North.—This Journal, January, &c.

——————————, by Mr. H. Cox.—Medical Repository, February.

Memoire sur les Causes des Convulsions chez les Enfans, &c. par M. Brachet. Paris, 1824.—Analysed in Anderson's Quarterly Journal, July.

An Essay on Tetanus, founded on Cases and Experiments, by Mr. Swann. London, 1825.—Analysed in this Journal, September.

Pathology of Tetanus, analytic article on.—Med. Repository, Sept.

Observations on Tetanus, by Mr. Ward. Gloucester, 1825.

Memoir of M. Marochetti on the Subject of Pustules under the Tongue in Hydrophobia.—Journal de Physiologie, Oct.; and this Journal, Dec.

Observations on Tic Douloureux, by Dr. Borthwick.—Edinburgh Med. and Surgical Journal, No. lxxxiii.

——————————, by Mr. Blackett.—This Journal, Oct.

Observations on Partial Paralysis, by M. Belladon.—Archives Generales, November 1824; Medical and Chir. Review, July 1825.

Case of Partial Paralysis of the Face, by Dr. Delafield.—New-York Med. and Phys. Journal, December 1824; Medical and Chirurg. Review, July 1825.

——— Paralysis, with Loss of Motion on one side and of Sensation on the other, by Mr. Dundas.—Edinburgh Med. and Surg. Journal, April.

——— Sleep continuing 451 Days.—Edinburgh Phil. Journal. April.

Cases of Hydrophobia.—Edinburgh Med. and Surgical Journal, Nos. lxxxiii. and iv.

—————————— Annali Universali, June; and in this Journal, September.

—————————— Medico-Chir. Transactions, vol. xiii. part i. London, 1825.

—————————— Medical Repository, May and July.

—————————— In this Journal, December.

—————————— not from the Bite of a Rabid Animal, by Dr. Whymper.—In this Journal, December.

—————————— Lancet, March 5, 19, May 28, August 27.

Bite of a Rabid Animal followed by Sublingual Pustules, by Dr. Baup, —Gazette de Santé; Lancet, October 15.

Case of Traumatic Tetanus.—Lancet, July 16, November 12, December 10.

——— Chronic Hydrocephalus.—Id. March 19.

Case of singular Spasmodic Affection of the Muscles of the Shoulder and Neck.—Id. July 16.

——— curious Morbid Affection of the Nerves.—Id. August 27.

——— Chronic Abscess in the Brain.—Id. April 9 and 16.

——— Epilepsy, attended with remarkable Slowness of the Pulse, by Dr. Burnett.—Medico-Chir. Transactions, vol. xiii. part i.

——— Epilepsia Sympathetica, in which a Portion of the Ulnar Nerve was removed.—Lancet, July 2.

——— Mental Imbecility, produced by Injury of the anterior Tibial Nerve, by Dr. James Anderson.—New-York Med. and Phys. Journal, No. 4; Medical and Chirurgical Review, April.

——— Phrenitis, followed by Enteritis, by Mr. Howell.—Medical Repository, September.

——— Meningitis, by Dr. Davies.—Id. April.

——— Spasmodic Affection of the Muscles of the Neck.—Lancet, May 14.

——— Hysterical Cough, by Dr. Sinclair.—Edinburgh Med. and Surg. Journal, January.

——— Disorganisation of the Spinal Marrow, giving rise to no Symptoms which tended to point out its Existence, by M. Andral.—Revue Med. Mars ; and in this Journal, May.

——— Inflammation of the Spinal Marrow, considered as the Cause of various Affections of the Chest and Abdomen.—Revue Med. February; Lancet, April 16.

——— Concussion of the Spine, tending to confirm the Opinion that the Nerves of Sensation and Motion are distinct, by Mr. Dundas.—Edinb. Medical and Surgical Journal, No. lxxxiii.

*Pathology of the Organs of Sense.*

Pathological State of the Eyeball and its Coats during the Course of the Ophthalmia of new-born Children, by Dr. Ammon, of Dresden.—Edinburgh Journal, No. lxxxiii.

Remarks on Iritis, by Dr. A. Robertson.—Id. No. lxxxii.

Suppuration of the Internal Ear, with Loss of the Bones, without Injury to the Sense of Hearing.—Gräfe and Walther's Journal für Chirurgie, band 7, heft 2; and in this Journal, December.

Cases of Destructive Inflammation of the Eye, by Dr. M. Hall and Mr. Higginbottom.—Medico-Chir. Transactions, vol. xiii. part i.: analysed in this Journal, December.

Case of Inflammation of the left Ear, caused by the presence of several Larvæ.—Journal der Praktischen Heilkunde: Lancet, January 8.

*Pathology of the Circulating System.*

A succinct Account of Researches made on Inflammation of the Veins, by M. Ribes.—Archives Generales ; Lancet, September 10.

Inflammation of the Veins, by Dr. Chapman.—Philadelphia Journal, February 1824; Medical and Chir. Review, April.

Instance of a Family affected with an hereditary Disposition to Hemorrhage.—Hufeland's Journal, Feb. 1824; Med. Repository, June.

Pericarditis, analytic article on.—Medical and Chir. Review, April.

On some Effects of the Loss of Blood, by Dr. M. Hall.—Medico-Chir. Transactions, vol. xiii. ; Medical Essays, London, 1825; and in this Journal, November.

Non-existence of Sugar in the Blood and Saliva of Diabetic Patients, by M. Vauquelin.—Revue Medicale, December 1824; in this Journal, Feb. 1825; Lancet, April 16.

History of a singular Case of Tendency to Plethora, by Dr. Musgrave. —Medical Repository, February.

Case of Disease of the Heart, by Dr. Baker.—This Journal, June.

——— Universal Pulsation of the Veins, by Dr. Berger.—Journal Complementaire, Juin; and in this Journal, August.

——— a Cicatrix found on the Heart.—Archives Generales, January; This Journal, March; Lancet, April 9.

——— a remarkable Disease of the Heart, by Dr. J. Johnson.—Med.-Chirurgical Transactions, vol. xiii. part i.

——— extensive Disease of the Heart, with Hydrothorax.—Lancet, November 26.

——— Rupture of the Vena Cava and Intestines.—Id. February 26.

Case in which the Blood was converted into a Matter resembling the Lees of Port Wine, by M. Velpeau.—Archives Generales, Mars; and this Journal, May.

*Pathology of the Absorbent System.*

Researches respecting the History of Diseases of the Lymphatic System, by M. Andral.—Medical Repository, February.

*Pathology of the Respiratory System.*

On Diseases of the Bronchia, Clinique Medicale, &c. by M. Andral. Deuxième partie. Paris, 1824.—Analysed in Med. Repository, Jan.

Intermittent Affection of the Chest.—Bibliotheque Medicale, July 1824; Med. and Chir. Review, July 1825.

Case illustrating the Pathology of Cynanche Laryngea.—Edinburgh Medical and Surgical Journal, No. lxxxii.

Cases of Fatal Dyspnœa, by M. Andral.—Revue Medicale, Sept. 1824; Medical and Chir. Review, April 1825.

Case of Fatal Asthma from an Affection of the Glottis, by M. Bouillaud. —Journal Comp. Juillet 1824; Medical and Chir. Review, April 1825.

Three Cases of Croup, with Observations tending to point out the contagious Nature of that Disease, by Dr. G. Gregory.—This Journal, Oct.

*Pathology of the Digestive System.*

On some Effects of Habitual Costiveness, by Dr. Borthwick.—Edinburgh Medical and Surgical Journal, No. lxxxii.

Pathology of the Digestive Mucous Membrane, analytic article on.—Medical Repository, October.

Remarks upon acute Dysentery.—Med. and Chir. Review, July.

On some Diseases of the Stomach, by M. Bourdon.—Revue Medicale, May 1824; Medical and Chir. Review, January 1825.

De l'Influence de l'Estomac sur la Production de l'Apoplexie, &c. by M. Richond. Paris, 1824.—Reviewed in Medical Repository, June 1825.

Remarks upon Obstructions of the Intestines, by Mr. Morley.—This Journal, October.

De la Membrane Muqueuse Gastro-intestinale, dans l'etat sain et dans l'etat inflammatoire, par M. Billard. Paris, 1825.—Analysed in this Journal, October; and Anderson's Journal, same month.

Observations on the Nature of Hepatic Ileus, by Dr. Musgrave.—Med. Repository, November.

On Obliteration of the Canal of the Strangulated Portion of Intestine, which has occasionally been produced by adhesive Inflammation of the Mucous Membrane, by Dr. Birkopp.—Idem.

On the Effects of Intestinal Irritation, by Dr. M. Hall. Medical Essays. London, 1825.

Case of Death from Inflammation of the Gall-bladder, occasioned by the Irritation of a Stone, by Dr. Scott.—Edinburgh Medical and Surgical Journal, No. lxxxiii.

Case of Larvæ of Insects found in the Human Stomach, by Dr. Gull.—Edinburgh Philos. Journal, July.

Cases of Obliteration of the Biliary Ducts, by M. Andral.—Archives Generales, October 1824; Medical and Chir. Review, July 1825.

Fatal Case of Hæmatemesis.—Revue Medicale, Mars; and Medical and Chir. Review, July.

Case of Ulceration and Rupture of the Stomach, by Dr. Elliotson.—Medico-Chir. Transactions, vol. xiii. part i. This Journal, October.

Excessive Secretion of Viscid Mucus in the Stomach.—Philadelphia Journal. This Journal, December.

Case of diseased Spleen, successfully treated, by Mr. Hartle.—This Journal, October.

——— Rupture of the Intestines and Vena Cava.—Lancet, February 26.

*Pathology of the Urinary System.*

An Inquiry into th Nature and Treatment of Diabetes, Calculus, and other Affections of the Urinary Organs, by Dr. Prout. London, 1825.—Reviewed in Lancet, July 2 and 16; Anderson's Quarterly Journal, Oct.

Practical Observations on certain Pathological Relations which exist between the Kidneys and other Organs of the Human Body, by Mr. Fosbroke. Cheltenham, 1825.—Reviewed in the Lancet, Sept. 3.

Existence of Mercury in the Urine of Patients under Mercurial Action, by Dr. Canton.—Memoires de Turin. This Journal, February.

Case of diseased Kidney, in which there were present only Symptoms of Hepatic Disease, by Mr. Tripe.—This Journal, September.

*Pathology of the Uterine System.*

History of a Case of Hydrometra, by Dr. A. T. Thomson.—Medico-Chir. Transactions, vol. xiii. part i. This Journal, December.

Barrenness in Prostitutes caused by the fimbriated Extremity of the Fallopian Tubes adhering to the neighbouring Parts.—Id. This Journal, October.

*Pathology of the Skin.*

Remarks upon Prorigo Formicans, by M. Alibert.—Nouvelle Bibliotheque Medicale, May; Medical Repository, October.

Anatomy, Physiology, and Pathology of the Skin, analytic article on.—Medical and Chir. Review, January.

Case of Ichthyosis.—Lancet, July 23, 30.

Severe Case of Lepra.—Id. September 10.

Case of Psoriasis Inveterata.—Id. July 30.

Fatal Cases of Purpura Hæmorrhagica.—Id. August 20.

Case of Rupia.—Id. September 17.

*Pathology of the Cellular Membrane.*

On Diffuse Inflammation of the Cellular Membrane, by Dr. David Scott.—Edinburgh Medical and Surgical Journal, No. lxxxv.

Case of Diffuse Inflammation of the Cellular Membrane, by Mr. Wiseman.—Id.

*Pathology of the Bones.*

An Arrangement and Description of the Diseases of Bones, by Dr. W. Cumin.—Edinburgh Medical and Surgical Journal, No. lxxxii.

Case of Fracture and Consolidation of the Clavicle in a Fœtus in Utero.—Archives Generales, March; Lancet, May 7. This Journal, Sept.

*Miscellaneous Points in Pathology.*

On the Malignant Pustule of Butchers.—Gräfe und Walther's Journal für Chirurgie, band 7, heft 2. This Journal, October.

On Exhaustion and Sinking from various Causes, by Dr. M. Hall. Medical Essays. London, 1825.

Remarks on Bronchocele, by Dr. A. Coventry.—New-York Medical and Phys. Journal, June 1824; Medical and Chir. Review, July 1825.

Effects of the Bite of the Tarantula, by Dr. Mazzolani, Osservatore Medico.—Lancet, October 15.

Observations on the Saliva during the Action of Mercury upon the System, by Dr. Bostock.—Medico-Chir. Transactions, vol. xiii. part i. This Journal, October.

On the Effects of Loss of Blood, by Dr. M. Hall.—Idem. This Journal, November.

Remarks upon the "Living Skeleton," by Mr. Boyle.—This Journal, Oct.

Pathology of Phlegmasia Dolens, by M. Velpeau.—Archives Generales, October 1824; Medical and Chir. Review, July 1825; Anderson's Quarterly Journal, October.

Observations on Phlegmasia Dolens, by Dr. Davies.—Med. Repos. July.

Peculiar Disposition to Closure of the Mouth.—Gräfe and Walther's Journal; Lancet, October 29.

Various Pathological Appearances observed in new-born Children, by M. Bricheteau.—Lancet, July 2.

Case of Inflammation of the Tongue.—Gräfe and Walther's Journal, band 7, heft 2. This Journal, December.

——— the simultaneous Occurrence of Small-Pox and Measles, by Mr. Delagarde.—Medico-Chir. Transactions, vol. xiii. part i.

——— Phlegmasia Dolens in a Male, by Dr. Davies.—Med. Rep. June.

——— Death from Acupuncturation.—Revue Medicale, Avril. This Journal, June.

——— a Burn, followed by great Cerebral Excitement.—Lancet, February 26.

### MEDICINE.

### *Medicine in general.*

The Study of Medicine, by Dr. Mason Good. Second edition. London, 1825.—Reviewed in this Journal, August; Anderson's Journal, October.

Elements of the Theory and Practice of Physic, by Dr. G. Gregory. Second edition. London, 1825.

A Nosological Practice of Physic, by Dr. G. P. Dawson. London, 1825.—Analysed in the Medical and Chir. Review, April.

A Compendium of Theoretical and Practical Medicine, by Dr. Uwins. London, 1825.—Analysed in Med. and Chir. Review; Lancet, Feb. 12, &c.

Lectures and Observations in Medicine, by the late Dr. Baillie. London, 1825.—This Journal, Nov. and Dec.; Med. Repository, December.

Art of detecting Diseases (in a series of articles.)—Medical Repository, March, &c.

Review of Medical Theories.—Idem, April, &c.

### *Particular Points in Medicine.*

An Account of the Diseases observed at the Hôtel Dieu, in the Clinical Wards of M. Recamier, during the first three months of the year 1825.—Lancet, June 25. This Journal, September.

Clinique Medicale, Maladies de Poitrine, by M. Andral.—Analysed in the Medical and Chir. Review, October.

Practical Remarks on Indigestion, particularly as connected with Bilious and Nervous Affections of the Head and other Parts, by Mr. Howship. London, 1825.—Reviewed in this Journal, November.

Observations on Gout, by Mr. Rennie. London, 1825.—Reviewed in the Medical Repository, December.

A Practical Treatise on Diabetes, &c. by Dr. Venables. London, 1825. —Reviewed in the Lancet, December 24.

On the Symptoms and Cure of Croup, by Mr. Mackenzie.—Edinburgh Medical and Surgical Journal, No. lxxxiii.

On the Treatment of Pertussis, by Dr. Venables.—This Journal, Sept.

On Chorea, with Cases to illustrate the Nature and Treatment of that Disease, by Dr. Jeffreys.—Edinburgh Med. and Surg. Journal, No. lxxxiii.

On Cauterisation of the Pustules in Small-Pox, M. Meyraux.—Archives Generales, September. This Journal, December. Lancet, Nov. 5.

Observations on the Treatment of Pulmonary Disorders, by Mr. Rennie. —Medical Repository, June.

Clinical Reports on Dropsies, &c. by Dr. Venables. London, 1824.— Reviewed in this Journal, February.

On Hydrophobia and its Treatment, by Professor Rust.—Reviewed in Edinburgh Medical and Surgical Journal, No. lxxxv.

Proposal of a Plan of Treatment for the Prevention of Hydrophobia, by Dr. Wendt.—Reviewed idem.

Canine Madness, or Hydrophobia, and the best means of preventing the occurrence of the Disease, by Dr. C. F. Lutheritz.—Reviewed idem.

Treatment of Persons bitten by mad Dogs, as practised in the Hospital at Zurich.—Hecker's Litterarische Annalen der Gesammten Heilkunde, June; Edinburgh Medical and Surgical Journal, No. lxxxv.

On the Treatment employed at the Hotel Dieu of Orleans, in Poisoning by Lead.—Archives Generales, March; Lancet, April 30.

New Method of treating Diseases produced by Lead, by M. Banque.— Archives Generales; and Med. Repository, July.

Observations on the Bite of the Viper, and on Ammonia as a Remedy, by Sig. Palletta.—Mem. dell' I. C. R. Instituto di Milano; Lancet, April 16.

On the Treatment of Scrofulous Diseases, by Dr. Venables.—Medical Repository, December.

Researches into the Nature and Treatment of Dropsy, by Dr. Ayre.— London, 1825.

Sur le Traitement des Tubercles du Poumon, par le Docteur Nasse.— Reviewed in the Medical Repository, September.

Recherches Nouvelles et Observations pratique sur le Croup, et sur la Coqueluche, &c. par M. Guibert. Paris, 1824.—Analysed in Anderson's Quarterly Journal, July.

An Introduction to the Use of the Stethoscope, with its Application to the Diagnosis in Diseases of the Thoracic Viscera, by Dr. Stokes.— Edinburgh, 1825.

A Treatise on the different Methods of investigating the Diseases of the Chest, particularly Percussion and the use of the Stethoscope; translated from the French of M. Collin, by Dr. Ryland.—1825.

Case of Chronic Hydrocephalus, successfully treated by Pressure, by Mr. Barnard.—Medical Repository, September.

Cases of Erysipelas, treated chiefly by Leeches and warm Fomentations, by Dr. Burrell.—Edinburgh Medical and Surgical Journal, No. lxxxv.

——— Tic Douloureux, successfully treated with Carbonate of Iron; communicated by Mr. Hutchinson.—This Journal, October.

Case of Tic Douloureux, relieved by Sulphate of Quina.—Lancet, January 22.

——— Traumatic Tetanus in a Horse, successfully treated by the Administration of Tobacco, by Mr. Egan.—This Journal, September.

——— Cataract and Gutta Serena, by Dr. Gondret.—Revue Medicale, June.—This Journal, September.

——— Hydrocephalus, successfully treated, by Dr. Henry Davies.— Medical Repository, March.

Case of Chronic Hydrocephalus, treated by Puncture, by Mr. Holbrook.—Idem, October.

——— Purpura Hemorrhagica, successfully treated by the evacuant plan, by Dr. Belcher.—This Journal, March.

——— Punctured Wound with Tetanus, successfully treated by Stimulants, by Dr. Nicholls.—Idem.

——— Diabetes cured by Bleeding, by Dr. Lefevre.—Journal de Physiologie; Lancet, April 2.

——— Poisoning by Oxalic Acid, successfully treated, by Mr. J. H. Wishart.—Edinburgh Medical and Surgical Journal, No. lxxxiv.

——— Tetanus, successfully treated, by Dr. G. Alexander.—Idem, No. lxxxv.

———————————, by Mr. W. Manfold.—Idem.

——— Purpura Hemorrhagica, successfully treated with Spirit of Turpentine, by Dr. I. Magee.—Idem.

——— Hemorrhoea Petechialis, successfully treated by Purgatives, by Dr. John Darwall.—Edinburgh Med. and Surg. Journal, No. lxxxii.

Cases illustrating the dangerous Effects of the Abuse of Iodine.—Idem.

Three Cases in which the Mammæ disappeared under the use of Iodine, by M. Hufeland.—Journal der Praktischen Heilkunde. This Journal, May.

Case of painful Affection of the Arm following Venesection, cured by Acupuncturation, by Dr. Webster.—This Journal, July.

Accidents arising from Acupuncturation.—Archives Generales, January. This Journal, March.

Case of Hydrocephalus cured by Mercury, by Dr. H. Fisher.—Edinburgh Medical and Surgical Journal, No. lxxxii.

——— Chorea, cured by rubbing Tartar-emetic Ointment into the Scalp, and along the course of the Vertebral Column, by Mr. R. Hunter.—Idem, No. lxxxiii.

Cases of Chorea, cured by Subcarbonate of Iron, by Dr. Elliotson.—Medico-Chirurgical Transactions, vol. xiii. part i. This Journal, Dec.

### *Materia Medica.*

Medical Researches on the Effects of Iodine in Bronchocele, Paralysis, &c. by Dr. Manson. 1825.—Extracts from, in this Journal, October: reviewed in Lancet, December 3.

Practical Observations on Mercurial Purgatives, by Dr. Gibson.—This Journal, July.

Observations on the Use of Colchicum in Gout, by Dr. Scudamore. London, 1825.—Reviewed in Lancet, March 26; Medical Repository, June. This Journal, July.

On the Use of the Rhus Toxicodendron in Palsy.—This Journal, July.

Codiate of Morphia, account of its Effects, by M. Olivier.—Idem, Dec.

Chloride of Lime, account of its Effects in Burns, by M. Lisfranc.—Archives Generales September. This Journal, Dec.

Case of Rheumatic Palpitation of the Heart, cured by Colchicum.—Lancet, December 10.

Comparative Experiments with Hyocyamia and Atropia on the Iris, by Dr. Reisenger, of Landshut.—Idem, September 17.

On the Nature and Use of the Thridace, or Juice of the Garden Lettuce.—Gazette de Santé, November 5; Lancet, December 10.

Bark of the Pomegranate Root a Remedy for Tænia.—Revue Medicale, November 1824. This Journal, July 1825.

Successful Employment of Muriate of Gold in Syphilis, by Dr. Benaben.—Lancet, December 3.

An Account of various Experiments performed in Italy with the Cinchona Bicolorata, by MM. Brera, Carminata, and Palletta.—Anderson's Quarterly Journal, October.

On the Use of the Croton Oil in Inflammation of the Bowels, by M. Morichini.—Annali Universali, December. This Journal, May.

Remarks on the Medicinal Effects of the Croton Oil, by Mr. Tegart.—This Journal, August.

On the Use of the Carob, by Mr. P. C. Blackett.—Idem.

On the Use of Nux Vomica in Palsy.—Journal General. This Journal, April.

On the Virtues of Black Pepper as a Febrifuge, by Dr. Clock, of Trent.—Giornale de Chirurgia Practica, March. This Journal, September.

Effects of the Chloride of Lime in Typhus.—Revue Medicale, June. This Journal, September.

On the Use of the Red Sulphate of Iron as an Astringent, by Dr. Bracconot.—Archives Generales, Juin. This Journal, September.

On the Flowers of Colchicum, by Mr. Frost.—Med. Repository, August.

On the Decoction of the Root of the Pomegranate in cases of Tape-worm, by Dr. Milne.—Medical and Chir. Review, October.

On the Action of Calomel on the Human Body, by Mr. Annesley.—In a work on the Diseases of India. London, 1825.

Materia Medica, Reviews upon.—Medical Repository, March, &c.

A Treatise on the Properties and Medicinal Application of the Vapour Bath, &c. by Dr. Gibney.—Analysed in the Lancet, August 27. This Journal, October.

On the Effects of a very hot and prolonged Bath in Chronic Rheumatism, by M. Feallier.—Journal Universel, Nov. 1824; Medical Repository. This Journal, February.

On the Employment of hot Water in Gout and Rheumatism, by M. Cadet-de-Vaux.—Lancet, June 4.

Oil of Euphorbia Lathyrus, a Substitute for Croton Oil.—Lancet, April 9; Medical and Chir. Review, July. This Journal, September.

Use of Iodine in Venereal Affections, by M. Eusebius de Salle.—Jour. Complementaire, Sept. 1824; Medical and Chir. Review, July 1825.

Caution against the Use of Stramonium.—Med. and Chir. Review, April.

On the Effects of Morphia and its Acetate, by M. Orfila.—Journal de Chimie, May; Medical Repository, July.

On the Medicinal Properties of Carbonate of Iron, by Dr. Elliotson.—Medico-Chir. Transactions, vol. xiii. part i. This Journal, Dec.

Two Cases of Tic Douloureux, cured by the external Application of Belladonna, by Mr. Henry.—This Journal, June.

Case of Poisoning by Prussic Acid, with Observations on Ammonia as an Antidote.—Revue Medicale, February; Lancet, April 23.

——— Pneumonia, treated with Tartar-emetic, by Dr. C. Anderson.—Edinburgh Medical and Surgical Journal, July.

Cases illustrating the Use of Cold Affusion to the Head in Poisoning by Opium.—New-York Med. and Chirurg. Journal; Anderson's Quarterly Journal, July.

---

### STATISTICAL MEDICINE.

The Art of Prolonging Human Life, by M. Hufeland.—Reviewed in this Journal, February.

An Account of the Disease lately prevalent at the General Penitentiary, Millbank, by Dr. P. M. Latham. London, 1825.—Analysed in Lancet, April 2 and 16; Medical Repository: this Journal, June. Medical and Chir. Review, and Anderson's Quarterly Journal, July.

Report of the Epidemic Cholera; as it has appeared in the Territories subject to the Presidency of Fort St. George, by Mr. W. Scott. Madras, 1824.—Analysed in this Journal, January; Medical and Chir. Review, and Edinburgh Journal, July.

Observations on the Cholera Morbus of India, by Dr. Whitelaw Ainslie. —Reviewed in Edinburgh Medical and Surgical Journal, July.

On Cholera, more especially as it has occurred during late years in British India, by Mr. T. Brown.—Reviewed in idem.

Elements of the Etiology and Philosophy of Epidemics, by Dr. Smith. New York, 1824.—Reviewed in Medical and Chir. Review, July.

Military Medical Reports; containing Pathological and Practical Observations, illustrating the Diseases of Warm Climates, by Dr. James M'Cave.—Reviewed in Medical Repository, April.

On Contagion (with a view of proving its non-existence), by Mr. Larkin. —This Journal, April, May, June.

Copy of a Dispatch from Sir Thomas Maitland, on the Subject of the Plague, (with a view of proving its contagious nature.)—This Journal, Sept.

On the Contagion of Yellow Fever, by Dr. Cherwin.—Bulletin des Sciences Medicales, Juin. This Journal, Sept.

A Letter to the Right Hon. W. Huskisson, M.P. on the Quarantine Bill, by Dr. Granville. London, 1825.

A Brief Sketch of the Progress of Opinion upon the Subject of Contagion, by Dr. Macmichael. London, 1825.

Fever, Contagion, Quarantine, an analytic Article on.—Medical and Chir. Review, January.

Drs. Omodei and Mars on Contagion.—Reviewed in the Edinburgh Med. and Surg. Journal, July.

Review of the Article upon Contagion and Sanatory Laws, contained in the Westminster Review.—Lancet, March 19 and April 30.

On the Natural and Medical Topography of the Western Part of Jamaica, by Mr. D. Mason.—Medical Repository, April.

Frequency of Goitres, especially in Women in the River District of the Paraiba.—Spix and Martius' Travels; Edinburgh Phil. Journal, April.

Recherches Statistiques sur la Durée moyenne des Fievres Intermittentes par M. Bailly.—Analysed in this Journal, November.

Histoire des Marais et des Maladies causées par les Emanations des Eaux Stagnantes, par M. Monfalcon. Paris, 1824.—Analysed in the Lancet, April 23; Medical and Chir. Review, October.

Medical Report of the Fever Hospital and House of Recovery, Cork-street, Dublin, for the year 1825, by Dr. Grattan. Dublin, 1825.—Reviewed in the Lancet, December 17.

On the Preservative Power of Belladonna over Scarlatina.—Journal der Praktischen Heilkunde; Lancet, April 2; Edinburgh Med. and Surgical Journal, No. lxxxii.

On Vaccination, by Dr. Shearman.—Medical Repository, February.

An Estimate of the true Value of Vaccination, by Mr. Greenhow.

Thoughts on Vaccination, and the Cause of its failing to afford the same Protection against Variola now, as formerly, by Mr. M'Ghie. Dumfries.

Proposal for a new Method of inoculating the Small-Pox, which deprives it of Danger, &c. by Dr. Ferguson. London, 1825.

Small-Pox and Cow-Pox; comprehending a concise History of those Diseases, by Mr. I. I. Cribb. Cambridge, 1825.

Observations on the Contagious Properties of the Varioloid Disease, or Modified Small-Pox, by Dr. Venables.—This Journal, June.

Small-Pox epidemic at Paris.—Lancet, Nov. 5, 12, and Dec. 17.

### MEDICAL JURISPRUDENCE AND MEDICAL POLICE.

Elements of Medical Jurisprudence, by Dr. Beck; edited by Mr. Dunlop. London, 1825.—Reviewed in Lancet, February 12; Anderson's Quarterly Journal, July.

———— Extracts from, on Feigned Diseases.—This Journal, May.

An Analysis of Medical Evidence, by Dr. Gordon Smith. London, 1825. —Medical and Chir. Review, and Anderson's Quarterly Journal, July; Medical Repository, March.

————, Extracts from, in this Journal, March.

On the Presence of Water in the Lungs of Drowned Persons, by M. Mayer.—Journal Complem. and Medical Repository, March.

Account of a Trial for Rape, taken from "Dissertazione Medico-Forense riguardante la Causa della Illmo. Sig. Achille Crespi, accusato di Stupro immaturo: autore L. Metaxa, &c. &c. Roma, 1824.—Anderson's Quarterly Journal, July.

Trial for Murder, and Pardon on the Plea of Insanity, by Dr. Beck.—New-York Med. and Phys. Journal; and idem.

Culpable Abortion, case of, by M. Foderé.—Idem, November 1824; and idem, April 1825.

Case of an Individual to whom a Drachm of powdered Cantharides was administered.—This Journal, December.

——— a Man who recovered after having swallowed half an Ounce of Cantharides, by M. Fontenelle.—Revue Med. Sept.; Lancet, Nov. 5.

Questions connected with Poisoning by Arsenic.—Gazette de Santé; Medical and Chir. Review, July.

Supposed Case of Poisoning by Arsenic, by M. Orfila.—Archives Gen. January; Lancet, May 12.

Case of Poisoning by Nux Vomica, with an Analysis of the Contents of the Stomach, by MM. Orfila and Barruel.—Idem, May; idem, July 16.

### SURGERY.

The Lectures of Sir A. Cooper, Bart.; edited by Mr. Tyrrell. Vol. ii. London, 1825.

An Essay on Venereal Diseases, and the Uses and Abuses of Mercury in their Treatment, by Mr. Carmichael. London, 1825.—Reviewed in the Lancet, April 30; in this Journal, May and June; and Medical and Chir. Review, October.

A Practical Treatise on Hæmorrhoids, or Piles, Strictures, and other important Diseases of the Rectum and Anus, by Mr. Calvert.

Observations on the Extraction of diseased Ovaria, by Mr. Lizars.—Reviewed in Lancet, Sept. 17.

A Short Treatise on the Section of the Prostate Gland in Lithotomy, with an Explanation of a safe and easy Method of conducting the Operation on the Principles of Cheselden, by Mr. Aston Key.—Reviewed in the Edinburgh Medical Journal, July.

On the Treatment of Wounds received during Dissection, by Mr. Shaw. —This Journal, February.

Method of Preventing the bad Effects of Wounds received in Dissection, by Dr. Godman.—Philadelphia Journal, No. 18. This Journal, June.

On Acupuncturation, by M. Pelletan, jun.—Revue Med. Juin; Archives Generales, Mars; Lancet, May 14; Medical and Chir. Review, July.

Method of employing Galvanism along with Acupuncturation, by MM. Bally and Meyraux.—Revue Medicale, October; Lancet, December 10.

History of a Case of Calculus in the Bladder, in which the Operation of Lithotomy was performed according to the method adopted by Mr. Aston Key; by Mr. C. Averill.—Edinburgh Med. and Surg. Journal, No. lxxxii.

Remarks on the Operation of Lithotomy, by Mr. Liston.—Idem.

A Treatise on Stricture of the Urethra, &c. by Mr. Macilwain. London.

Extirpation of the Tonsils, Mr. Pettigrew on.—Med. Repository, May.

Wound of the Carotid Artery, by M. Delpech.—Revue Medicale, Dec. 1824; Medical and Chir. Review, July 1825.

On Amputation of the Hip-joint, by M. Walther; with an answer to his Strictures on Mr. Guthrie's Operation.—Med. and Chir. Review, Oct.

On the Treatment of Scrofulous Ulcerations, by Mr. Rennie.—Medical Repository, March.

On the Treatment of Ophthalmia by Nitrate of Silver, by Dr. Ridgway. —This Journal, February.

A Short Treatise on Operative Surgery, by Mr. Averill. Cheltenham, 1825.

Observations on the Treatment of Cataract, by M. Gondret.—Journal de Physiologie; and Medical Repository, October.

Description of an Instrument for destroying a Stone in the Bladder, by Mr. Griffiths.—Anderson's Quarterly Journal, October.

M. Civiale's Lithontriptor.—Revue Med. Juin; Lancet, July 9; this Journal, and Anderson's Quarterly Journal, October.

On the Healing of Wounds from Burns and Scalds, by the Scabbing Process, by Mr. Bush.—This Journal, September.

On the Use of the Syphon in removing the Contents of the Stomach.—Edinburgh Journal of Science, July.

On the Evils of Procrastinating the Operation in certain cases of Strangulated Hernia, by Mr. T. W. Chevalier.—This Journal, June.

Remarks on the Treatment of incised Wounds, by Mr. Symes.—Edinburgh Med. and Surg. Journal, No. lxxxiv.

On Amputation, by Mr. A. Dewar.—Idem, No. lxxxv.

Description of an Instrument for compressing the Inguinal and Subclavian Arteries, by Mr. C. Blackett.—This Journal, July.

———————————————— Carotid Artery, by Mr. C. Blackett.—This Journal, May.

Further Observations on Lateral or Serpentine Curvature of the Spine, by Mr. Shaw; London, 1825, (Extracts from).—This Journal, June, &c.

Carl Wenzel der Heilkunde Doctor, &c. über die Krankheiten am Ruckgrathe—Analysed in this Journal, April.

On Œsophagotomy, by M. Berlinghieri.—Med. Repository, April.

An Essay on the Extraction of Calculi from the Urinary Bladder, by Mr. W. Thompson. Edinburgh, 1825.

On Gastrotomy, by Mr. Waller.—This Journal, March.

An Essay on Curvature and Diseases of the Spine, by Mr. Bampfield.—Analysed in this Journal, March.

A Treatise on Moxa, as applicable more particularly to Stiff Joints, by Mr. Boyle. London, 1825.—Analysed in Medical Repository, March; Lancet, March 5; Med. and Chir. Review, April.

Illustrations of Acoustic Surgery, by Mr. Buchanan. London, 1825.

New Method of treating Compound Fractures, by Baron Larrey.—Journal Complementaire, Jan.; Med. and Chir. Review, July.

Practical Observations on Hydrocele, by Mr. Holbrook. London, 1825. —Reviewed in Lancet, June 25.

Remarks on the Diagnosis and on the Inversion of the Foot in Fracture of the Neck and upper part of the Thigh-bone, by Mr. Guthrie.—Medical and Chir. Transactions, vol. xiii. part i. This Journal, November.

Additional Observations on the Treatment of certain severe Forms of Hemorrhoidal Excrescence, by Mr. Kirby. Dublin, 1825.

Observations on the Dangers liable to arise from the less frequent use of the Trephine, by Mr. Wise.—Medical and Chir. Review, April.

The Science of Surgery, by Mr. Sleigh. London, 1825.

Practical Commentaries on the present Knowledge and Treatment of Syphilis, by Mr. Wellbank. London, 1825.—Reviewed in Anderson's Quarterly Journal, October.

A Full Account of the System of Friction in Cases of Lameness, &c. by Mr. Cleobury. Oxford, 1825.—Reviewed in Lancet, Feb. 5.

Operation for Intus-susception, by Dr. Fuschstius.—Hufeland's Journal, February; Medical and Chir. Review, October.

Operation of Lithotomy,* which proved fatal from Hemorrhage.—Lancet, November 12.

Operation of tying the Subclavian Artery.—Lancet, September 3.

Operation for Closing the Cleft Palate, by M. Roux.—Archives Generales; Lancet, July 2.

Operation for Imperforate Anus, by M. Dupuytren.—Revue Med. May; Lancet, July 30.

Operation for artificial Pupil.—Lancet, December 3.

Partial Amputation of the Hand, by M. Benaben.—Revue Med. March; Med. and Chir. Review, July.

Removal of a large Portion of the Scapula, by M. Janson.—Archives Generales, August. This Journal, October.

Cæsarean Operation, successfully performed, in a Case of Extra-uterine Pregnancy of some years' standing, during which period the Woman became pregnant in the usual way, and was safely delivered, by M. Ruth.—Gräfe and Walther's Journal; Edinburgh Med. and Surg. Journal, April; Lancet, May 7; Med. and Chir. Review, July.

Case of Cæsarean Operation, by Dr. Schonberg.—Idem, and idem; also Medical Repository, July.

——————————, in which the Mother and Child were saved, by Dr. Vanderfuhr.—Revue Med. October; Lancet, Dec. 10.

Case of Extirpation of the Clitoris, by which Idiocy of many years' standing was cured.—Gräfe and Walther's Journal der Chirurgie, subenter band. This Journal, July. Medical and Chir. Review, October; Lancet, December 17.

Successful Case of High Operation for the Stone, by Mr. Copland Hutchison.—Quarterly Journal of Science, Oct. This Journal, Nov.

Case in which a new Method of performing the Lateral Operation of Lithotomy was practised with success, by Mr. J. Wilkinson.—Edinburgh Medical and Surgical Journal, No. lxxxii.

Case of Staphyloma Sclerotica, successfully treated by repeatedly tapping the Eye, by Dr. B. Maitland.—Idem.

——— Fracture of the Os Pubis, with Separation at the Symphysis and Sacro-iliac Junction.—Lancet, April 16.

——— Fracture of both Sides of the Cranium, with Depression, by Mr. Neilson.—Edinburgh Medical and Surgical Journal, No. lxxxiii.

——— Compound Dislocation of the Ankle-joint.—Lancet, Jan. 29.

Successful Excision of the Labia Pudendi, enormously enlarged by Elephantiasis, by Dr. W. Borrel.—Idem.

Case of Cancer of the Uterus, cured by M. Baudelocque.—Archives Generales, Juin. This Journal, September.

---

* Of this operation, an account, by Mr. Shaw, is contained in the present Number of this Journal.

Extirpation of the Uterus, two cases of, from the German Journals.—Edinburgh Med. and Surg. Journal, April; Med. and Chirurgical Review, July.

Case of Extirpation of the Uterus, by Dr. Holscher.—Gräfe and Walther's Journal; Edinburgh Medical and Surgical Journal, April; Lancet, April 30; Anderson's Quarterly Journal, July.

——— Phlegmasia Dolens requiring Amputation, by Mr. Davies.—Medical Repository, June.

——— Ophthalmia cured by Acupuncturation, by M. Cloquet.—Revue Medicale, Mars. This Journal, May.

——— Compound Dislocation of the Knee, by Messrs. Muller and Hoffman.—Medical Repository, October.

——— Anchylosis of the Joints of the Lower Jaw, by Mr. Snell.—Med. Repository, February.

——— large Urinary Calculus extracted from the Female Bladder, by Dr. Mac Intosh.—Edinburgh Med. and Surg. Journal, July.

——— Fistula communicating between the Urethra and Vagina, occurring after Parturition, successfully treated, by Mr. Hobart.—This Journal, December.

——— a double Psoas Abscess, by Mr. Lizars.—Edinburgh Med. and Surgical Journal, No. lxxxiv.

Case in which the Operation for Artificial Anus was successfully performed, by Dr. R. Maitland.—Idem, No. lxxxv.

——— Congenital Hydrocephalus, in which the Operation of Puncturing was repeatedly performed, by Mr. James Sym.—Idem.

——— severe Compound Fracture, with Dislocation of both Ankle-joints, successfully treated, by Mr. J. Mitchell.—Idem.

——— Irritation of the Urethra and Neck of the Bladder, arising from Stricture of the Rectum, by Mr. White.—This Journal, July.

——— Diseased Larynx, in which Tracheotomy was performed, by Mr. W. Goodeve.—Idem.

——— Fracture of the Frontal Bone, with Escape of a Portion of Brain, successfully treated without Operation, by Dr. F. Corban.—This Journal, March.

——— Axillary Aneurism, successfully treated by tying the Subclavian Artery, by Mr. Key.—Medico-Chir. Transactions, vol. xiii. part i. This Journal, October.

——— Carotid Aneurism, successfully treated by tying the Artery above the Aneurismal Tumor, by Mr. Wardrop.—Idem, and Idem.

Cases in which the Operation for the Removal of Cicatrices from the Neck, consequent on Burns, was successfully performed, by Mr. James.—Idem.

——— Wound of the Parietes of the Abdomen, with a Protrusion of a large Portion of Intestine, successfully treated, by Mr. Dix.—This Journal, December.

——— Ruptured Tendon of the Patella, with Remarks, by Mr. Thompson.—Medical Repository, September.

Cases of Foreign Bodies lodged in the Trachea.—Gräfe and Walther's Journal, band 6, heft 2; Anderson's Quarterly Journal, July.

Case of Fractured Ribs, with Wound of the Lungs.—Lancet, Dec. 17.

——— Rupture of the Intestines, produced by violent Contusion of the Abdomen.—Idem.

——— Necrosis of the Bones of the Leg.—Idem.

——— long-continued Insensibility from Injury of the Head, in which it was deemed necessary to inject Fluids into the Stomach, by Mr. Oswald.—This Journal, November.

——— Traumatic Tetanus.—Lancet, January 29.

Case of Compound communated Fracture of the Tibia and Fibula.—Lancet, January 8 and 22.

Cases of Concussion.—Lancet, January 15, 22, 29, June 18, July 16, 23; September 3, October 15, 29.

——— Femoro-popliteal Aneurism.—Lancet, January 29, August 13.

Case of Inguinal Aneurism.—Lancet, June 25.

——— Axillary Aneurism.—Lancet, August 13, October 29.

——— a spontaneous Cure of Aneurism of the right Subclavian Artery, by M. Bernardin de Vezelay. — Archives Generales, Decembre 1824; Lancet, February 5.

——— Wound of the right Carotid Artery cured by frequent Bleedings, &c. &c. by M. Delpech.—Revue Medicale, Dec.; Lancet, Feb. 19.

——— Strangulated Femoral Hernia.—Lancet, March 26, July 2.

——————— Inguinal Hernia.—Id. June 11, 18, July 2, Oct. 22.

——————— Abdominal Hernia.—Id. September 17.

——— Scrotal Hernia.—Id. July 9, 30, August 13, September 17, and November 26.

——— Encysted Hernia of the Tunica Vaginalis.—Idem.

——— Inflammation of the Arm from Blood-letting.—Id. April 2.

——— Injury of the Spine.—Id. May 7, October 22.

——— Fractured Dorsal Vertebræ.—Id. September 17.

——— Fatal Wound of the Trachea.—Id. May 21.

——— Penetrating Wound of the Chest, by M. Toulmouche.—Revue Medicale, Mars; Lancet, June 4.

——— Fractured Spine.—Lancet, June 4.

——— Stricture of the Œsophagus.—Id. June 18.

——— Retention of Urine, in which the Bladder was punctured.—Id. October 29.

Cases of Retention of Urine, with Extravasation.—Idem, July 23, Sept. 10, October 15, November 26.

Case of Compound Fracture of the Tibia and Fibula.—Idem, Sept. 3.

——————————————————Idem, October 15.

——— Amputation at the Shoulder-joint.—Idem, September 10.

——— diffused Aneurism of the Thigh.—Idem.

——— Extirpation of the Uterus, by M. Laud Wolff.—Gräfe and Walther's Journal; Lancet, October 22.

——— Fatal Injury of the Head.—Lancet, October 29.

——— Compound Fracture of the Leg.—Idem.

——————————————————.—Idem, November 5.

——————— Dislocation of the Ankle-joint.—Idem.

——— Permanent Contraction of the Fore-arm succeeding Venesection.—Idem.

——— Fracture of the Os Frontis and Bones of the Face; Rupture of the Abdominal Muscles.—Idem.

——— apparently slight Injury of the Head, which proved fatal at the end of three weeks.—Idem, December 10.

——— Hemorrhage following Amputation.—Idem.

---

### MIDWIFERY.

Elements of Operative Midwifery, with a Description of certain new and improved Powers for assisting difficult and dangerous Labours, by Dr. D. D. Davis.—Reviewed in Lancet, July 30, Aug. 13; Anderson's Journal, Oct.

A Compendious System of Midwifery, by Dr. Dewees.—Reviewed in this Journal, July and August.

Obstetric Studies; comprehending a Treatise on Parturition, &c., by Mr. James Hogben.

Instructions to Mothers and Nurses on the Management of Children, in health and disease, by Dr. Kennedy.—Analysed in the Med. and Chir. Review, October.

On a new Variety of extra-uterine Pregnancy, by Dr. Gilbert Breschet. —Med.-Chirurgical Transactions, vol. xiii. part i. This Journal, October.

Observations on Transfusion of Blood; with an Account of two Cases of Uterine Hemorrhage in which that Operation has been recently performed with success, by Mr. Waller. London, 1825.

Cursory Observations on Preternatural Presentations, accompanied with a Case of unfrequent occurrence, by Mr. Brown.—Med. Repository, Jan.

On Puerperal Irritability, by Mr. Waller.—This Journal, February.

On the Secale Cornutum, (Cases illustrative of its utility.)—This Journal, July and August.

Case of a Fœtus in which the left Foot was separated from the Leg during Utero-gestation, by Mr. Watkinson.—This Journal, July.

——— a Child crying while the Head was still in Utero.—Archives Generales, January. This Journal, March.

——— Rupture of the Perineum, by Mr. Bond.—Medical Repository.

——— Fallopian-tube Pregnancy, by Dr. Elliotson.—Medico-Chirurg. Transactions, vol. xiii. part i. This Journal, October.

——— Rupture of the Uterus, by Dr. Broyles.—Philadelphia Journal, No. 17; Medical Repository, June.

——— Uterine Hemorrhage, in which the Operation of Transfusion was successfully performed, by Mr. Waller.—This Journal, October.

———————————————, by Mr. Doubleday.—Id. Nov.

——— true Uterine Pregnancy, supposed to have lasted upwards of three years, by M. Penker.—Allg. Medic. Annalen, September 1824; Anderson's Quarterly Journal, October 1825.

——— Twins, in which one Child was expelled seventeen days before the other, by Mr. Bush.—This Journal, February.

———Uterine Hemorrhage, suppressed by large Doses of Nitre.—Bull. de l'Athenée de Medecine; Lancet, May 21.

——— Rupture of the Uterus, with Escape of the Foetus into the Abdominal Cavity, Operation, and Recovery of the Mother, by Dr. L. Frank.—Annali Universali, January and February; Lancet, June 18.

### CHEMISTRY AND PHARMACY.

Elements of Medical Chemistry, by Dr. Paris. London, 1825.—Reviewed in this Journal, July; Anderson's Quarterly Journal, July and Oct.

A Popular Explanation of the Elements and General Laws of Chemistry, by Mr. Weldon. London, 1825.

An Attempt to establish the first Principles of Chemistry by Experiment, by Dr. Thomson. London, 1825.

A Manual of Pharmacy, by Mr. Brande. London, 1825.—Analysed in Med. and Chir. Review, July.

Experiments to ascertain how far the presence of Albumen and Muriatic Acid interfere with the action of Protiodide of Mercury and Protomuriate of Tin upon each other, by Dr. Bostock.—Edinburgh Med. and Surgical Journal, No. lxxxii.

Analysis of the Upas Tieute and Upas Antiar.—Idem.

Detection of Hydrocyanic Acid in the bodies of Animals poisoned by it, by M. Laussaigne.—Ann. de Chimie, No. 27; Quarterly Jour. of Science, January; Med. Repository, February.

M. Chansorel's new Chemical Doctrine.—Reviewed in Med. Rep. July.

Academical Examinations on the Principles of Chemistry, by Mr. Reil. —Edinburgh, 1825.

An Essay on the Natural, Chemical, and Medical History of Water; with a List of all the principal Mineral Waters in Europe, by Dr. Ryan.—This Journal, December.

Observations on the Tests for Arsenic, by Dr. Giseke.—Schweigger's Journal, band 13; Philosophical Magazine, October.

Method of detecting Spots of Blood, by M. Lassaigne.—Revue Med. Avril. This Journal, June. Anderson's Quarterly Journal, July.

On the Formation of Hydriodate of Potassa, by Dr. Ed. Turner.—Edinburgh Medical and Surgical Journal, No. lxxxiv.

On the Analysis of Opium, by Mr. T. Jeston.—This Journal, August.

On Pariglina, the alkaline principle obtained from Sarsaparilla.—Lancet, February 5. This Journal, April.

Mr. Donovan's Apparatus for filtering Fluids liable to be injured by exposure to the atmosphere.—Dublin Philosophical Journal, April; Medical Repository, October.

Facts towards the Chemical History of Mercury.—Quarterly Journal of Science, January. This Journal, February.

On the Impurity of the pulverised Emetic Tartar of the shops.—This Journal, July.

On the advantageous Preparation of the Ammoniacal Compounds.—Ann. de Chimie, xxviii.; Quarterly Journal of Science, July.

On a new Preparation of Croton Tiglium, by the late Mr. Pope.—Med.-Chir. Transactions, vol. xiii. part i. This Journal, October.

Opium, new Salt (Codiate of Morphia) discovered in, by M. Robinet; with an account of its effects on animals.—Journal de Chimie, &c. October. This Journal; and Medical Repository, December.

On the Preparation of Morphia, by M. Hottot.—Journal de Pharmacie. This Journal, February.

Remarks upon the active Principle of Belladonna, by M. Runge.—Ann. de Chimie, xxvii. 32. This Journal, February.

——————————— Colocynth, by M. Vauquelin.—Journal de Pharmacie. This Journal, February.

Preparation of Lactucarium, by M. Roman.—Revue Medicale, Aout. This Journal, November.

——————— Sulphate of Quina, by M. Caventon.—Archives Generales, Juillet. This Journal, November.

---

### MISCELLANEOUS.

An Essay on Egyptian Mummies, with Observations on the Art of Embalming among the ancient Egyptians, by Dr. Granville.—Philosophical Transactions, 1825. This Journal, August.

Method of preserving Bodies by injecting them with Whiskey, by Dr. Godman.—Philadelphia Journal, No. 18. This Journal, June.

Description of the general Hospital at Munich.—Nye Hygœa. This Journal, July.

On the Principles and Practice of Ventilating and Warming Buildings, by Mr. Tredgold.—Edinburgh Philosophical Journal, January and April.

Observations on Medical Education; being a Review of the Introduction to Dr. Paris's Medical Chemistry.—This Journal, May.

Description of an Hermaphrodite, by Dr. Mayer, of Bonn.—Lancet, October 22.

---

On future occasions we hope to render our LIST more complete. Gentlemen desirous of having their works, &c. inserted, are requested to send them to the Publisher.—EDITORS.

